# Cathodoluminescence Spectroscopy in Graded In_x_Ga_1−x_N

**DOI:** 10.3390/nano12213719

**Published:** 2022-10-23

**Authors:** Xiaofang Zhao, Tao Wang, Bowen Sheng, Xiantong Zheng, Li Chen, Haihui Liu, Chao He, Jun Xu, Rui Zhu, Xinqiang Wang

**Affiliations:** 1 School of Materials Science and Engineering, Tiangong University, Tianjin 300387, China; 2 Electron Microscopy Laboratory, School of Physics, Peking University, Beijing 100871, China; 3 State Key Laboratory for Mesoscopic Physics and Frontiers Science Center for Nano-Optoelectronics, School of Physics, Peking University, Beijing 100871, China; 4 Beijing Goldenscope Technology Co., Ltd., Beijing 100190, China

**Keywords:** full-composition-graded In_x_Ga_1−x_N films, cathodoluminescence, nitride semiconductor, paired defects

## Abstract

InGaN materials are widely used in optoelectronic devices due to their excellent optical properties. Since the emission wavelength of the full-composition-graded In_x_Ga_1−x_N films perfectly matches the solar spectrum, providing a full-spectrum response, this makes them suitable for the manufacturing of high-efficiency optoelectronic devices. It is extremely important to study the optical properties of materials, but there are very few studies of the luminescence of full-composition-graded In_x_Ga_1−x_N ternary alloy. In this work, the optical properties of full-composition-graded In_x_Ga_1−x_N films are studied by cathodoluminescence (CL). The CL spectra with multiple luminescence peaks in the range of 365–1000 nm were acquired in the cross-sectional and plan-view directions. The CL spectroscopy studies were carried out inside and outside of microplates formed under the indium droplets on the InGaN surface, which found that the intensity of the light emission peaks inside and outside of microplates differed significantly. Additionally, the paired defects structure is studied by using the spectroscopic method. A detailed CL spectroscopy study paves the way for the growth and device optimization of high-quality, full-composition-graded In_x_Ga_1−x_N ternary alloy materials.

## 1. Introduction

III-nitride semiconductors and their alloys have received widespread attention due to their excellent optical and electrical properties. Among the nitride semiconductors, InGaN alloys are widely used in optoelectronic devices. In addition, by adjusting the composition of indium (In), the bandgap of InGaN alloys can be continuously adjusted from 0.64 eV to 3.4 eV, covering a continuous spectrum from near-infrared to ultra-violet (UV), and is an ideal material for devices emitting white light [1,2,3]. At the same time, InGaN material has a high optical absorption coefficient and a relatively high electron saturation drift rate. The emission wavelength of the full-composition-graded In_x_Ga_1−x_N material perfectly matches the solar spectrum, which provides a full-spectrum response and is suitable for the manufacture of high-efficiency photoelectric devices [4,5,6]. However, due to the poor crystal quality of InGaN materials, the efficiency of InGaN-based photoelectric device materials is severely limited [7,8,9]. The high balance vapor pressure and growth temperature difference between InN and GaN make it difficult to obtain high-quality InGaN materials, especially for growing full-composition InGaN ternary alloy. A thin film of full-composition-graded In_x_Ga_1−x_N ternary alloy has been successfully grown by molecular beam epitaxy (MBE), and several research groups have reported epitaxial growth of related full-composition ternary alloy (Miller [10] and Zheng [11]). To improve the quality of the InGaN ternary alloy, two-dimensional stepwise growth has been adopted [12], and epitaxial growth is usually carried out under In-rich conditions and using growth temperature-controlled epitaxy [13]. Therefore, when growing a full-composition-graded In_x_Ga_1−x_N ternary alloy, In droplets were often formed on partial surfaces [14,15,16,17,18]. The full-composition-graded In_x_Ga_1−x_N ternary alloy can be applied in photoelectric devices and other fields. The study of the optical properties of In_x_Ga_1−x_N ternary alloy is extremely important, but reports on this are rare.

Here, we studied the optical properties of a full-composition-graded In_x_Ga_1−x_N ternary alloy film using cathodoluminescence (CL) measurements with a high spatial resolution and variable exciting electron beam. There are multiple luminescence peaks in the range of 365–1000 nm. Detailed CL spectroscopy was carried out inside and outside of the microplates, which formed under the indium drops after etching on the surface, and it was found that the emission intensity inside and outside of the microplates differs significantly.

## 2. Materials and Methods

The growth process of full-composition-graded In_x_Ga_1−x_N ternary alloy was reported [11]. Measurements of the high-angle annular dark-field scanning transmission electron microscopy (HAADF-STEM) were carried out using the aberration-corrected Thermo Fisher Scientific Titan Cubed Themis G2 transmission electron microscope operated at 300 kV. Morphology was characterized by ThermoFisher Quattro ESEM. CL spectra were obtained using Gatan Monochrome CL3+, a high-resolution CL probe with panchromatic and monochromatic reception at 300–800 nm wavelengths, and Rainbow-CL system with a spectral detection range of 300–1000 nm.

## 3. Results

### 3.1. Composition and Structure of the Full-Composition-Graded In_x_Ga_1−x_N film

**Figure 1:**
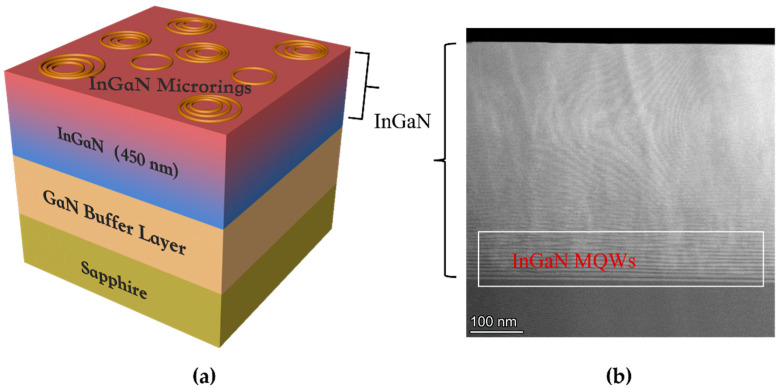
(**a**) Schematic illustration of the full-composition-graded In_x_Ga_1−x_N. (**b**) HAADF-STEM image of the full-composition-graded In_x_Ga_1−x_N.

The full-composition-graded In_x_Ga_1−x_N ternary alloy gradually passes from GaN to InN, that is, x goes from 0 to 1 in the In_x_Ga_1−x_N ternary alloy. The scheme of the sample structure is shown in Figure 1a. A 450 nm-thick full-composition-graded In_x_Ga_1−x_N film was grown on GaN buffer layer by the MBE. The cross-sectional specimen was prepared by mechanical polishing and argon ion milling. Figure 1b is a HAADF-STEM image of an InGaN layer with multiple periodic quantum well (MQW) striking structures in the initial region of an In_x_Ga_1−x_N film. The high-In (light) and low-In (dark) In_x_Ga_1−x_N segments are composed of MQW-like structures that promote effective strain relaxation [19]. The formation of a periodic structure is associated with the relaxation of stress between InN and GaN with a large lattice mismatch (~11%) [20]. Most parts of the full-composition-graded In_x_Ga_1−x_N ternary alloy contain MQW-like structures, where with the decrease in growth temperature and the change in the growth mode, the atomic diffusion length decreases, and the periodical ordered structure disappears.

### 3.2. Detailed Spectral Analysis of the Full-Composition-Graded In_x_Ga_1−x_N film

**Figure 2:**
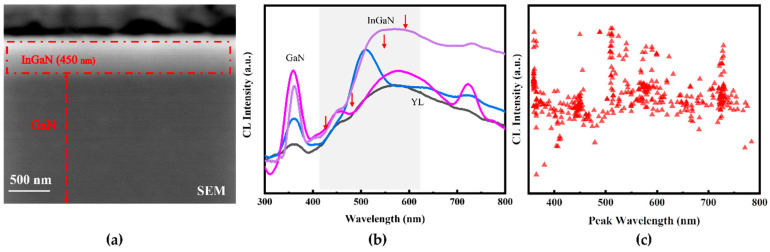
(**a**) Cross-sectional SEM image of the full-composition-graded In_x_Ga_1−x_N film. (**b**) CL spectra of the full-composition-graded In_x_Ga_1−x_N film under a beam voltage of 5 kV at room temperature. Four spectra were taken, respectively, from four random regions in the dotted red frame in Figure 2a. The main CL peaks of InGaN have been represented by arrows. (**c**) The wavelength distribution diagram of the CL luminescence peaks of the full-composition-graded In_x_Ga_1−x_N film under a beam voltage of 5 kV at room temperature.

To figure out the luminescence characteristics of quantum wells, CL measurements with high spatial and spectral resolution were used to accurately measure the luminescence of graded In_x_Ga_1-x_N film. The luminescence of graded In_x_Ga_1-x_N film in cross-sectional and plan-view were recorded at various accelerating voltages at room temperature. Figure 2a is a SEM image of a full-composition-graded In_x_Ga_1−x_N film in a cross-sectional direction. As shown by the red dashed-line box, the 450-nm-thick layer at the surface is a graded In_x_Ga_1-x_N layer.

The CL spectra of the cross-sectional samples derived from four randomly selected regions in the red frame in Figure 2a for a 5 kV electron beam, which are shown in Figure 2b. The CL spectra of each random region were obtained by continuous scanning of a 2.5 × 0.5 µm^2^ area. One can see the edge emission of the GaN band at 365 nm and a broad peak related to deep-level emission at 565 nm (the so-called yellow band), which is most commonly attributed to defects associated with gallium vacancies (V_Ga_) or defects associated with carbon [21,22]. Peaks between GaN and YL can be identified as the luminescence from the full-composition-graded In_x_Ga_1−x_N. As shown in Figure 2b, there are many irregular peaks, and broad peaks were found in the 400~800 nm range, proving the existence of luminescence in the entire visible spectral range.

In order to better understand all the luminescence peaks of a full-composition-graded In_x_Ga_1−x_N ternary alloy film, CL spectra were collected with a 5 kV electron beam, and the wavelength distribution diagram of the luminescence peaks of the full-composition-graded In_x_Ga_1−x_N ternary alloy film was extracted at room temperature (Figure 2c). The full-composition-graded In_x_Ga_1−x_N ternary alloy film continuously emits light in the range from 365 nm to 800 nm. Theoretically, for the full-composition-graded In_x_Ga_1−x_N ternary alloy film, the luminescence peak should be measured in the range of 365 nm~1850 nm. Due to the detection ability of our detector, the luminescence peak can only be seen in the range of 365–800 nm, which is further evidence that the full-composition-graded In_x_Ga_1−x_N ternary alloy film has great potential to cover the entire solar spectrum. There are some differences in the distribution of luminescence peaks. The intensity of the peak is high in the range of 400 nm~480 nm, 510 nm~600 nm, and 700 nm~750 nm, indicating strong luminescence in these wavelength ranges.

**Figure 3:**
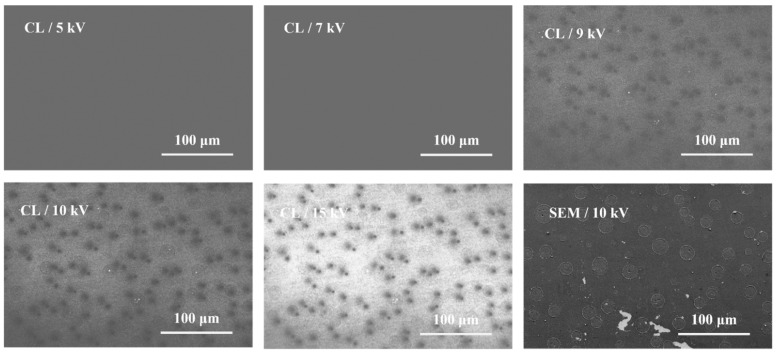
CL images of full-composition-graded In_x_Ga_1−x_N film excited with electron beams ranging from 5 kV to 15 kV at room temperature.

The lateral distribution of optical properties of full-composition-graded In_x_Ga_1−x_N ternary alloy was studied. As shown in Figure 3, different excitation voltages ranging from 5 kV to 15 kV were explored. Many microplates were spontaneously formed on the surface of full-composition-graded In_x_Ga_1−x_N films. Due to the degradation of crystal quality in the high-In composition region, the luminescence is weak at a low excitation voltage below 8 kV, and it is difficult to see a clear structure feature from CL images. With the increase in excitation voltage, the excitation depth of the electron beam can reach the low-In composition region. The crystal quality of these regions is high, so the CL signal and images are noticeable. However, since the In composition inside the microplates is higher than in other structures, the microplates could be observed more accurately at appropriate low voltages. The sizes of these In-rich microplates are not uniform, with the smallest microplate diameter being about 3 μm, while the largest microplate diameter is about 18 μm. The density of microplates is about 1.0 × 10^5^ cm^−2^, which is similar to the defects density of 1.23 × 10^5^ cm^−2^. We found that microplates have two different shapes: one is a typical “three-rings structure”, and the other is a “one-ring structure”. In addition, there is a different luminescence intensity in each microplate, which may be related to nonuniform In distribution. We also found that the defect structures always appeared in pairs near the microplates, that is, a circular defect structure with a diameter of about 3 μm and a dark spot structure with a diameter of about 5 μm. The luminescence intensity and In composition of the paired defects were also different. In order to verify the source of the paired defects, the CL spectrum analysis on the GaN buffer layer was conducted to exclude the possibility of a V-shaped defect in GaN. The density of the defects on the GaN buffer layer is approximately 7.9 × 10^7^ cm^−2^, which is much higher than the paired defects density of full-composition-graded InGaN and lower than the V-shaped defect density of GaN (see Supplementary Materials).

**Figure 4:**
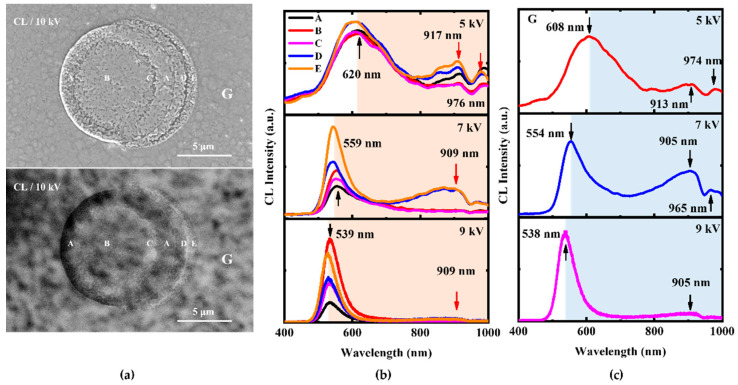
(**a**) SEM and CL images of full-composition-graded In_x_Ga_1−x_N film. (**b**,**c**) Their CL spectra of different structural positions (A–E, G) were obtained at 5 kV, 7 kV, and 9 kV beam voltages. The main CL peaks in Figure 4b,c have been indicated by arrows.

The luminescence characteristics of microplates and defect structures under different excitation voltages were further studied, as shown in Figure 4. There is a very clear CL spectral difference between the inside microplates (A–E) and the outside microplates (G). We conducted chemical wet etching for indium droplets. The indium droplets disappeared, leaving multiple “microplate” structures on the surface (see Supplementary Materials). The CL image shows that the overall luminescence intensity inside the microplates gradually decreases from the center (B) to the outer circle. Therefore, under the excitation voltage of 5 kV, the emission peaks (620 nm, 917 nm, and 976 nm) were observed in the spectra (Figure 4b).

CL spectra of microplates outside (G) were obtained in Figure 4c, the luminescence peaks of microplates outside are mainly at 608 nm under the excitation voltage of 5 kV. Compared with the microplate (Figure 4b), the peak position of microplates outside was lower at 5 kV, and the composition of indium in microplates outside (Point G) is slightly lower than in microplates inside (Point B). Meanwhile, the CL luminescence intensity of microplates outside is the highest compared with other structures, and the crystal quality of microplates outside is the best, which is the main luminescence structure of the full-composition-graded In_x_Ga_1−x_N film. This indicated that the content of In in the inner microplate is higher than outer surface, an increase In content will lead to the deterioration of crystal quality [18]. Namely, as the In content increases, the quality of the crystal at B is worse than at G, so the quality of the microplates inside is worse than the microplates outside. This experimental result is also consistent with the corresponding theoretical analysis mentioned above. We believe that these differences can be associated with dislocations and uneven distribution of In composition in the film of the In_x_Ga_1−x_N ternary alloy.

**Figure 5:**
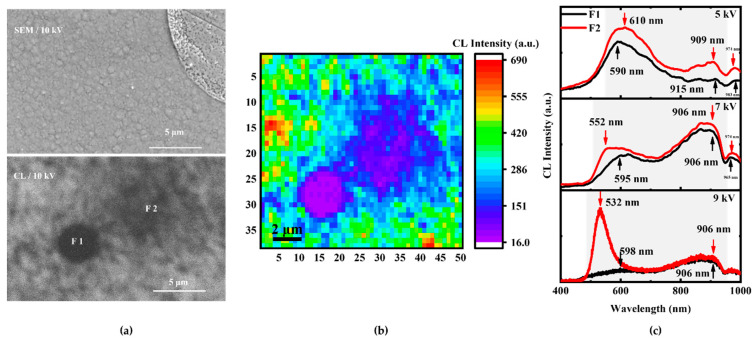
(**a**) SEM and CL images of the paired defects (F1 and F2). (**b**) CL mapping image of the paired defects at 10 kV beam voltages. The scanning area contains 50 × 38 pixels. (**c**) CL spectra of the paired defects at 5 kV, 7 kV, and 9 kV beam voltages. The main CL peaks have been indicated by arrows.

Combined with CL images and CL spectra, we believe that the paired defects (F1 and F2) in Figure 5 could be caused by the following two factors: (1) Because the stable luminescence peak at various excitation voltages, the F1 point may be related to surface clustered penetration dislocations or other defects, which may stem from the GaN and InGaN interfaces or the InGaN layer, and the crystal quality is the worst. (2) During the growth process of full-composition-graded In_x_Ga_1−x_N film, the composition of In increased gradually with the growth process, while the crystal quality decreased, which may further generate partial short threading dislocations. In this process, the F2 point appears, and the peak position of CL spectra is mainly determined by the In composition. It was more direct to observe the correlation between the F1 point and F2 point for their crystal quality through CL mapping in Figure 5b. As a rule, the lattice and thermal mismatch between the epitaxial layer and substrate cause threading dislocations, in which nonradiative recombination centers reduce the quantum efficiency of light-emitting devices [17,23,24,25]. As the excitation voltage increases, the CL luminescence peaks of the F2 point move in the direction of the short wavelength. We found that the outline of the peak of the F2 point includes the F1 point in Figure 5c, which is related to the electron beam of interaction volume overlapping with the F1 and F2 points [26].

### 3.3. CL Mapping Observation of InGaN Microplates and Full-Composition-Graded In_x_Ga_1−x_N Film

**Figure 6:**
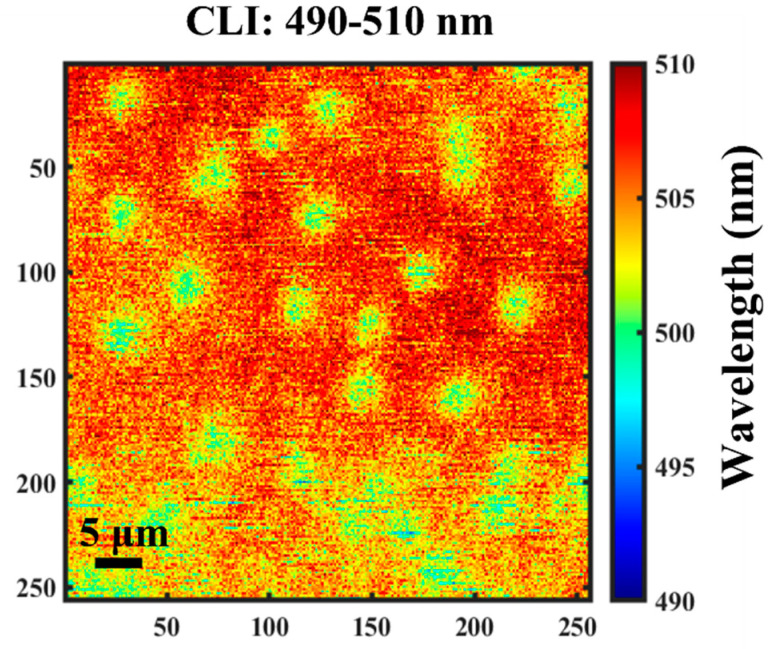
Two-dimensional mapping of full-composition-graded In_x_Ga_1−x_N film under 15 kV excitation voltage with the wavelength range from 490 nm to 510 nm at room temperature.

CL measurements with high spectral and spatial resolution were performed to elucidate the lateral homogeneity and emission properties of InGaN at room temperature. Due to the inhomogeneous distribution of the In composition, some luminescence peaks can be concentrated in certain wavelength regions, which is related to fluctuations in the In composition during growth. Figure 6 shows a-two-dimensional mapping image of InGaN with the CL wavelength ranging from 490 nm to 510 nm. The luminescence peaks of InGaN at 15 kV are mainly around 510 nm, while the luminescence peaks of paired defects are mainly around 503 nm. In addition, the luminescence wavelength of the two structures is not identical, with a difference in luminescence wavelength of about 7 nm.

There is a large lattice mismatch between the InGaN and the GaN buffer layer. The strain caused by lattice mismatch has a great influence on the growth process of InGaN, especially the composition of indium. This phenomenon is called the composition-pulling effect [27], which makes it difficult to grow highly crystalline InGaN with a high In content on GaN. The incorporation of indium was inhibited by compressive strain but enhanced by tensile strain [28]. Thread dislocations penetrate the InGaN layer and reach the InGaN surface where the defects are formed. The formation of defects weakens the lattice deformation and the pulling effect of the composition [29], which is beneficial to the incorporation of indium, which leads to the composition of indium change—that is, the wavelength of the CL luminescence, which goes toward the long wavelength. The composition-pulling effect, strain, and defects decide the nonuniform In distribution.

The growth mechanism of InGaN microplates has been extensively studied [16,18,24,30,31,32,33,34,35], while the CL properties of full-composition-graded In_x_Ga_1−x_N film are rarely reported, especially InGaN microplates. We found that the CL luminescence intensity of microplates inside was significantly weaker than microplates outside. This also indicates that the distribution of In components inside and outside of the microplates is not uniform. The composition of In in the In_x_Ga_1−x_N microplates inside was higher than in InGaN microplates outside because more indium atoms were accumulated in the “ring”. Compositional fluctuations in the area of microplates are related to the process of growth microplates. In addition, we also found a sharp change in the light intensity at the edge of a single microplate, which may be due to the high efficiency of doping with absorbed indium atoms [18]. These results are also related to the surface energy of the In_x_Ga_1−x_N alloy [36].

## 4. Conclusions

In this work, the luminescence characteristics of a full-composition-graded In_x_Ga_1−x_N film are studied by SEM-CL. The results showed that visible luminescence peaks of the InGaN quantum structure were observed around 447 nm, 530 nm, 550 nm, 600 nm, 620 nm, 920 nm, and 976 nm. The full-composition-graded In_x_Ga_1−x_N ternary alloy film continuously emits light in the range from 365 nm to 1000 nm, which shows that the In_x_Ga_1−x_N materials have a promising spectral response with the solar spectrum. In addition, we analyzed the CL properties of InGaN microplates and paired defects on the surface of In_x_Ga_1−x_N films. InGaN microplates can effectively control the CL luminescence characteristics of In_x_Ga_1−x_N ternary alloy. These structures have a significant effect on the optical properties of In_x_Ga_1−x_N films. The discovery of multi-wavelength luminescence peaks provides important ideas for the study of material growth and structure research of the full-composition-graded In_x_Ga_1−x_N ternary alloy.

## Data Availability

The data presented in this study are available on request from the corresponding authors.

## Supplementary Materials

The following supporting information can be downloaded at: https://www.mdpi.com/article/10.3390/nano12213719/s1, Section S1: CL spectra of full-composition-graded In_x_Ga_1-x_N at low temperature; Section S2: CL spectrum of GaN buffer layer; Section S3: Excitation depth, In composition, and luminescence wavelength of full-composition-graded In_x_Ga_1-x_N film at different excitation voltage; Section S4: In droplet structure on the full-composition-graded In_x_Ga_1−x_N before etching. References [37,38] were cited in the Supplementary Materials.
