# Supplementary material for: Cathodoluminescence Spectroscopy in Graded InxGa1−xN
Source: Nanomaterials (Basel). 2022 Oct 23;12(21):3719. doi: 10.3390/nano12213719 (PMC9658634; doi:10.3390/nano12213719)
Supplement: Supplementary file 1 [file nanomaterials-12-03719-s001.zip › nanomaterials-1919890-supplementary.pdf]

Article

Cathodoluminescence Spectroscopy in Graded $In_xGa_{1-x}N$

Xiaofang Zhao [1], Tao Wang [2,*], Bowen Sheng [3], Xiantong Zheng [3], Li Chen [2], Haihui Liu [1,*], Chao He [4], Jun Xu [2], Rui Zhu [2,*] and Xinqiang Wang [3]

[1] School of Materials Science and Engineering, Tiangong University, Tianjin 300387, China
[2] Electron Microscopy Laboratory, School of Physics, Peking University, Beijing 100871, China
[3] State Key Laboratory for Mesoscopic Physics and Frontiers Science Center for Nano-Optoelectronics, School of Physics, Peking University, Beijing 100871, China
[4] Beijing Goldenscope Technology Co., Ltd., Beijing 100190, China
* Correspondence: cwwangtao@pku.edu.cn (T.W.); liuhaihui@tiangong.edu.cn (H.L.); zhurui@pku.edu.cn (R.Z.)

This file includes:

S1. CL spectra of full-composition-graded $In_xGa_{1-x}N$ at low temperature.

S2. CL spectrum of GaN buffer layer.

S3. Excitation depth, In composition, and luminescence wavelength of full-composition-graded $In_xGa_{1-x}N$ film at different excitation voltage.

S4. In droplet structure on the full-composition-graded $In_xGa_{1-x}N$ before etching.

S1. CL spectra of full-composition-graded InxGa1-xN at low temperature.

CL spectra were measured at low temperature from 300 K to 80 K, which was shown in Figure S1). The peak positions move to lower wavelength with decreasing temperature (Figure S1(c)). The temperature dependence of semiconductor band gaps is explained by Varshni relation:

$$E_g(T) = E_0 - \alpha T^2/(T + \beta)$$

where fitting parameters α and β are related to given material. Which agree well with our experiment data.

At the same time, with the decrease of temperature, the integral intensity increases (Figure S1(d)). The nonradiative transitions will be suppressed at low temperature [Materials Science and Engineering B-advanced Functional Solid-state Materials, **1996**, 42 : 24-31; Materials Sciences and Applications, **2014**, 5 : 267-270]. And the number of electron-hole pairs available for radiation recombination increase due to the weakened thermal activation at low temperature [Journal of Physics: Condensed Matter, 2004, 16: S279-S285], which also increase the CL intensity. There were two strong emission peaks of $In_xGa_{1-x}N$ at 370 nm and 500 nm at 83 K, respectively.

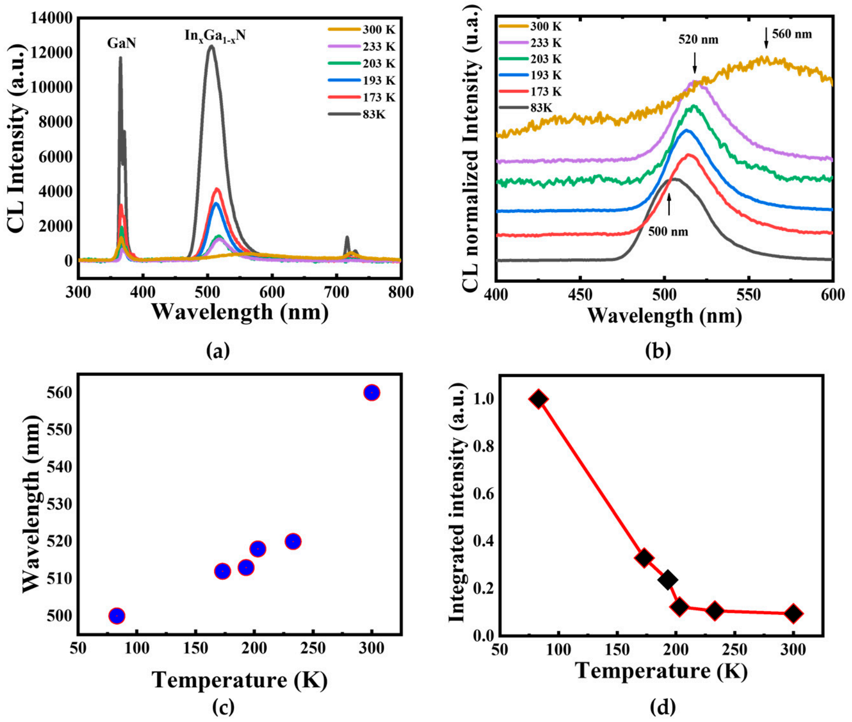

Figure S1. CL spectra were measured at low temperature. (**a**) Cross-sectional CL spectra from 300-800 nm of the full-composition-graded $In_xGa_{1-x}N$ film. (**b**)Normalized intensity of cross-sectional CL spectra in the 400-600 nm band. (**c**) The CL emission wavelength from 400 nm to 600 nm as a function of temperature. (**d**) The integral CL intensity change with temperature from 400 nm to 600 nm. The electron beam was 10 kV.

S2. CL spectrum analysis on the GaN regrowth layer of graded $In_xGa_{1-x}N$ film.

In order to verify the source of the paired defects, we conducted CL spectrum analysis on the GaN buffer layer to exclude the possibility of V-shaped defect in GaN. The density of the defects on GaN buffer layer is 7.9×10^7 cm^{-2}, which is much less than the V-shaped defect density of GaN. Therefore, we believe that the paired defects of graded $In_xGa_{1-x}N$ film are related to threading dislocation bundles.

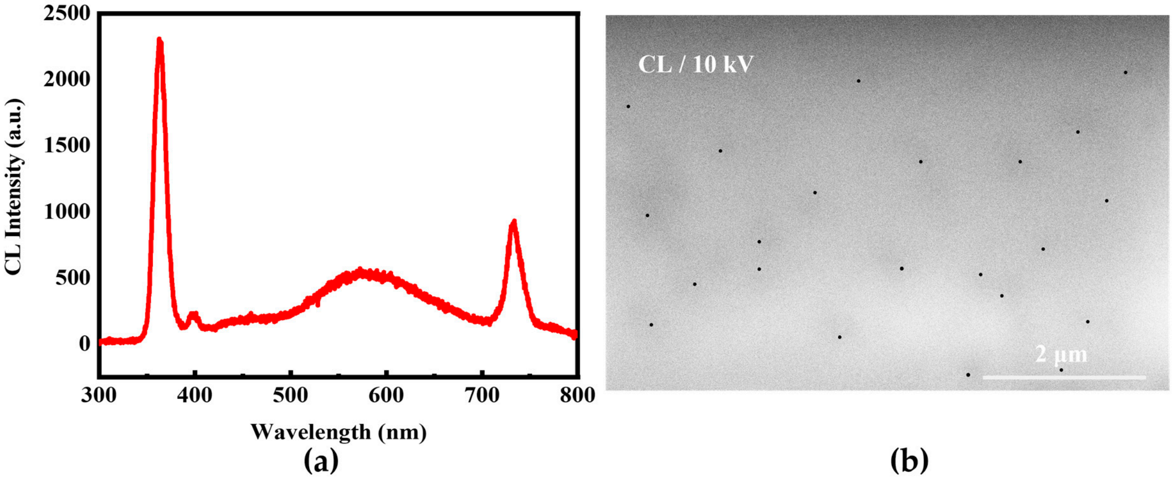

Figure S2. (**a**)CL spectrum and CL imgae (**b**) of GaN buffer layer at room temperature under 10 kV electron beam.

S3. Excitation depth, In component, and luminescence wavelength of full-composition-graded $In_xGa_{1-x}N$ film change with different excitation voltage.

Because of the difficulty in Monte Carlo simulation for full-composition-graded $In_xGa_{1-x}N$, we estimated the excitation depth and other relevant data of the full-composition-graded $In_xGa_{1-x}N$ based on the Monte Carlo simulation for $1n_{0.255}Ga_{0.745}N$ layer and $In_{0.17}Ga_{0.73}N$ [MRS Online Proceedings Library, 1997, 482: 738-741; Physical Review B, 2001, 64: 205311], and EDS line scan results we have done before for full-composition-graded $In_xGa_{1-x}N$ [Applied Physics Letters, 2020, 117: 0021811].

At an excitation voltage of 3 kV, $In_{0.255}Ga_{0.745}N$ layer was excited to a depth of about 90 nm, while $In_{0.17}Ga_{0.73}N$ was excited to a depth of about 83 nm. This means that the penetration depth of the electron beam will increase with the increase of the In composition. Therefore, the electron beam penetration depth of the full-composition-graded $In_xGa_{1-x}N$ layer should be larger than $In_{0.255}Ga_{0.745}N$ layer under the same excitation voltage, because the paired defects and poor crystal quality of full-composition-graded $In_xGa_{1-x}N$, which is also consistent with our experimental results. This also proved that we observed a wavelength of 620 nm under excitation of 5 kV electron beam, which is theoretically only visible under 6 kV beam voltage.

Table S1. Excitation depth, In composition, and luminescence wavelength of full-composition-graded $In_xGa_{1-x}N$ film change with different excitation voltage.

Voltage (kV)	Excitation depth (nm)	In composition	Wavelength (nm)
3	90[1]	0.76[2]	1290.04[3]
5	200[1]	0.60[2]	968.40[3]
7	350[1]	0.23[2]	501.96[3]
9	480[1]	0.00[2]	-
11	760[1]	0.00[2]	-

S4. In droplet structure on the full-composition-graded $In_xGa_{1-x}N$ before etching.

The surface of graded $In_xGa_{1-x}N$ film before and after chemical wet etching is shown in Figure S3. Figure S3a is the indium droplet structure without etching, microplates formed after chemical wet etching was shown in Figure S3b. The indium droplets have disappeared, leaving only some multiple "microplate" structures on the surface.

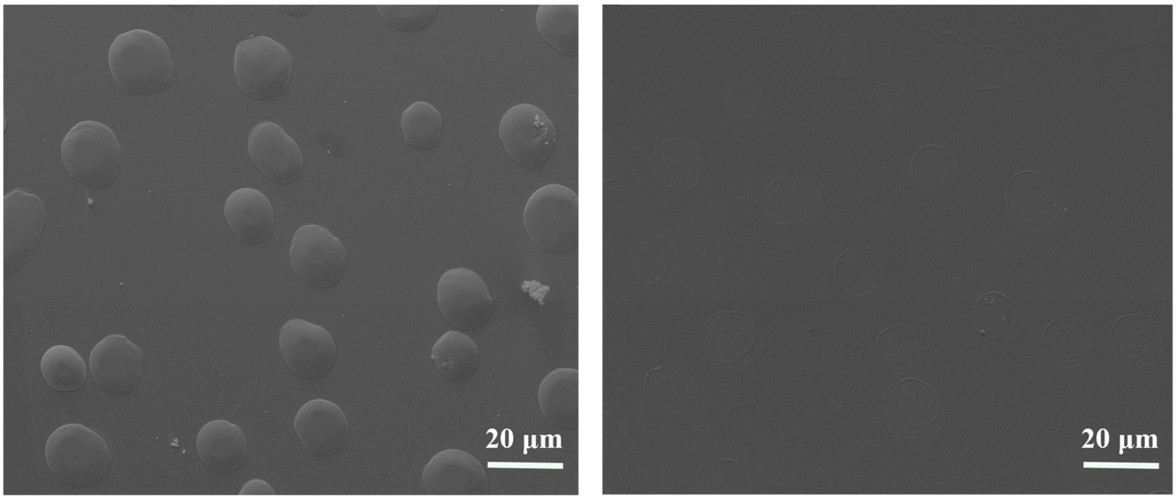

(a) (b)

Figure S3. SEM images of the graded $In_xGa_{1-x}N$ surface grown by MBE. (**a**) Before and (**b**) after chemical wet etching.

1. Trager-Cowan, C.; Middleton, P.G.; Mohammed, A.; O'Donnell, K.P.; Van der Stricht, W.; Moerman, I.; Demeester, P. Probing the Indium Mole Fraction in an InGaN Epilayer by Depth Resolved Cathodoluminescence. *MRS Online Proceedings Library* **1997**, *482*, 738-741, doi:10.1557/PROC-482-715.
2. Zheng, X.T.; Wang, T.; Wang, P.; Sun, X.X.; Wang, D.; Chen, Z.Y.; Quach, P.; Wang, Y.X.; Yang, X.L.; Xu, F.J.; et al. Full-composition-graded InxGa1−xN films grown by molecular beam epitaxy. *Applied Physics Letters* **2020**, *117*, doi:10.1063/5.0021811.
3. McCluskey, M.D.; Van de Walle, C.G.; Romano, L.T.; Krusor, B.S.; Johnson, N.M. Effect of composition on the band gap of strained InxGa1−xN alloys. *Journal of Applied Physics* **2003**, *93*, 4340-4342, doi:10.1063/1.1560563.
